# Age, ageing, and the philosophy of ‘elder law’: An interview with Israel (Issi) Doron

**DOI:** 10.1007/s41020-022-00176-7

**Published:** 2022-12-06

**Authors:** Ankita Gandhi

**Affiliations:** Humboldt University, Berlin, Germany

**Keywords:** Jurisprudential gerontology, Ageism, Older adults, Domestic abuse and abandonment, Ageivism, COVID-19

## Abstract

Gerontology—the multidisciplinary study of the process of ageing and its medical and social consequences for older adults—has often not been given its due by either legal practitioners or legal academics. While we have witnessed several social movements advocating for the rights and interests of other marginalised groups, discrimination against older adults has also not been the subject of any mass action comparable to those movements. Consequently, the position of older adults within legal systems has frequently been bypassed, especially outside the United States of America. However, the COVID-19 pandemic suddenly brought the precarious position of older adults into the spotlight, be it in care homes during lockdowns or in hospitals through several waves or with regard to priority access to vaccines. At each juncture, governments took recourse to legal regulation to mitigate the impact of the disease on what they understood to be the most vulnerable sections of the population. But some of these rules were subjected to criticism, especially from older adults themselves, because of their overbreadth, and on account of the protectionism and infantilisation of older adults that they entailed. In this interview, Professor Israel (Issi) Doron, Dean of the Faculty of Social Welfare and Health Sciences of the University of Haifa, Israel, discusses how academia, strategic litigation, and advocacy and activism have finally begun taking the field of ‘jurisprudential gerontology’ into serious consideration.

## Introduction

The several draconian lockdowns that the Government of India imposed to curb the spread of COVID-19^1^ compelled me to move back to my parents’ house, or what I now call my childhood home, and stay with them for an uninterrupted nine months. My father turned 70 in November 2020, in the midst of the pandemic, and spent his birthday (and the subsequent two weeks) in quarantine, after having tested positive. Those two weeks were perhaps the most difficult of the entire pandemic for me, since I had been convinced by the hysteria whipped up by mass media that the disease was akin to a death sentence for the so-called elderly.^2^ He was perhaps more terrified than I was, being a more avid consumer of doomsaying news coverage than I could ever be. Yet, he remained asymptomatic when I, younger than him by 41 years, struggled with long COVID for weeks. A representative of the Government of the National Capital Region of Delhi would ring his cell phone the first couple of days, telling us that the government’s personnel would have to *compulsorily*^3^ isolate him in their medical facility and keep him under constant observation. The stress of being forcibly separated from his family at such a tense hour could have potentially caused more damage than the disease itself.

He would contract COVID-19 once again a year and a half later and remain *miraculously* unharmed once more. There are several older adults like him, who have easily lived to tell the tale. But is it fair to label cases such as his ‘miraculous’? What this near panic about the impact of COVID-19 masks is the differences in the socio-economic status (such as race, poverty, gender, and health parameters) amongst older adults that determine mortality rates from COVID-19 more than just age.^4^ It is the adamant refusal by states to remove this homogenising veil that has led to older adults being isolated far more than other social groups, causing their mental health to be impacted in the most detrimental of ways.^5^

This incident also brings into focus the role of the welfare state in supporting older adults. The Indian state does not have a great track record of providing adequate social security—in the form of old-age pensions and state-sponsored medical assistance—to its older citizens,^6^ even though Article 41 of the Constitution of India provides for ‘public assistance’ in cases of ‘old age’.^7^ American jurist Martha Albertson Fineman has, in explaining the concept of social security for older adults, delineated a distinction between the ‘negative’ and ‘positive’ assumptions of old age that provide the basis for social security policies.^8^ Pensions and medical assistance for older adults can be said to have originated partly from the ‘positive assumptions’ that older adults are entitled to these benefits based on the contributions they have made to society and the economy in their ‘productive years’ (the upper limit of which is often stipulated by the law through the age of superannuation).^9^ These assumptions contribute to greater autonomy for older persons. But some policies, like requiring older adults to undergo mandatory hospitalisation during the pandemic without giving them a general right to medical assistance under all circumstances, flow solely from ‘negative assumptions’ associated with advanced age, namely vulnerability, and physical and mental incapacity. This assumption of vulnerability culminates in the ‘monitoring, disciplining, and supervision’^10^ of older adults, and, instead of recognising them as rights bearing entities, strips them of their dignity and leaves them to the mercy of the state. Fineman adds,

If someone is very young, profoundly ill or disabled, or very old, we may not be comfortable demanding they conform to the mandates of self-sufficiency and independence. They are perceived as needing protection, and paternalism guides society’s response—which is to withhold agency, as is the case with children, or take away agency based on assumptions about lack of capacity, as we do with many of the elderly.^11^

With pandemic-related restrictions being relaxed from mid-2021, I moved to Berlin to pursue my PhD, giving me another reason to take a deeper interest in ‘law and gerontology’, especially in what Professor Doron believes is a crucial aspect of the ‘international dimension’ of ageing—‘distant caring’ of older adults by their children who have emigrated to other jurisdictions, and the legal hassles involved in the ‘reunion of families’ once they are in separate countries.^12^ I then joined a Facebook group named *Indians in Berlin*^13^ where people seek help in navigating complicated German bureaucratic processes. On the one hand, several Indians often express a desire to have their dependent older parents join them in Germany; however, it is next to impossible for them to emigrate.^14^ On the other hand, although the Residence Act in Germany allows an immigrant with a residence title to leave German territory for six months,^15^ employers and funding agencies can impose more restrictive conditions. For instance, I could easily visit my parents in February 2022 for nine weeks after they had just about recovered from their second bout of COVID-19 in India’s third wave,^16^ but when my PhD grant commences from 01 October 2022, I will not be able to visit them for more than six weeks. My funding agency will discontinue my stipend and health insurance (on which my residence permit depends) if I decide to be away from Germany for more than six weeks.^17^ This stipulation is far more stringent than the one imposed by the German government, even though the timelines of an unstructured PhD like mine do not have to coincide with German universities’ academic calendars.

With my personal experiences forming the backdrop of many of the questions I wanted to pose, I spoke to Professor Doron over Zoom on 31 July 2022 about his work, and discussed its relevance for my own academic and personal dilemmas. In this interview, he charts his journey as an academic and activist, discusses the development of the field of ‘law and ageing’ (also known as ‘elder law’) and his own contribution towards giving shape to the legal philosophy of gerontology, explains his suggestions for the future of the rights of older persons, and the fissures within movements that endeavour to combat ageism.

I have annotated the interview with detailed citations to help the reader follow and look up the incidents, events, works, and concepts discussed herein.

## Interview

### Ankita Gandhi (AG):

*Several academics eschew the use of the words ‘elderly’ or ‘senior’ for older adults, arguing that they tend to homogenise the differences in physical and psychological status—conveying an oversimplified and generalised sense of frailty, vulnerability, and dependence—amongst a diverse set of people, socially and legally grouped together only by an arbitrary chronological age-marker.*^18^*There has been a shift in the terms that you have been using as well. Would you agree that terminology, in and of itself, can have an ageist impact? An 80-year-old can run a marathon*,^19^*while in a different circumstance a 70-year-old can perhaps need a care home. Do you think that terminology, in this context, can be ageist?*

### Israel Doron (ID):

The short answer is—yes. I truly agree and believe that the terminology we use reflects our biases, stereotypes, and social constructions. With other similarly disadvantaged groups in society — for instance, women or persons with disabilities—terminology is a part of their human rights struggles in general. Within the scientific community also there is a struggle to change terminology, as part of changing the perceptions, attitudes, and constructions of these groups. We have a similar struggle, a valid struggle, to change the terminology in the scientific world with regard to older persons or older adults. I must admit that while English is not my first language, I still do understand the notion that the words ‘elderly’ or ‘senior’ are, on the one hand, more related historically to vulnerability and to dependency. They can also, on the other hand, reinforce the stereotypical privileged perceptions of older adults.^20^ So, the transition to using a more neutral term or moving to the terminology of using ‘older persons’ or ‘older adults’ is, I think, true and correct. I may add that this is true not only in English; however, I can only testify for my mother tongue Hebrew, where we also have various words describing older persons [that may be ageist].^21^ Once again, many of these words historically were connected to frailty, dependency, etc., and we have moved, both in the general society, and in the scientific and legal terminology, towards a more neutral, less stigmatising and historically less dependency- or frailty-oriented terminology. This is a part of political and social struggles to raise awareness about the rights of older persons and to combat ageism. I definitely agree and support such a movement and to adopt a non-ageist terminology in the field of gerontology.

### AG:

*On the question of terminology, in an article from 2012*^22^*and a blogpost in 2013*,^23^*you have mentioned two distinctive phrases—‘jurisprudential gerontology’ and ‘geriatric jurisprudence’. How are these divergent from the more commonly used concepts of ‘elder law’ and ‘law and gerontology’? Was there something in particular at that time, in terms of context, that led you to focus on these two terms instead of ‘elder law’ or ‘law and gerontology’, because unfortunately, even though you’ve said terminology is important, these terms still haven’t caught on as much as one would like. I was looking at the LSE library and I barely got a handful of resources on ‘jurisprudential gerontology’ or ‘geriatric jurisprudence’, most of which were written by you.*^24^

### ID:

First of all, I believe all things take time, hence the adoption of new concepts and theories needs time in order to be picked up and applied in research, science, and policy.

I also think that one needs to make some differentiations between terms and their historical context. The term ‘elderly’, that you mentioned in the previous question, is a broad word used often in various disciplines. It could be in sociology, gerontology, or psychology. This is a word that crosses disciplinary lines and borders, and the movement to change the term or stop it from being used is universal and general. The concepts you have just mentioned like ‘elder law’ or ‘law and ageing’ or ‘therapeutic jurisprudence’ or ‘jurisprudential gerontology’ are all much more specific to the field of law and ageing.

Historically, the field of law and ageing originated in the USA, and it was practice-oriented within the country. So, it grew bottom-up, from the field, from practitioners and lawyers in the field who started to develop an area of specialisation which reflected their target audience or clientele, meaning older persons who were the clients of private lawyers and attorneys who addressed their specific legal issues. Hence, it was termed ‘elder law’. In the American context, the mention of ‘elder law’ included Medicare or Medicaid entitlements,^25^ which is a very America-specific health-care insurance concept for older persons, or it was seen during estate planning,^26^ which is again a very North American tax law–specific area.

When I originally entered the field, and as I wrote in some of my articles, I did so through my experience during my stay in the US. As an Israeli, I think this [i.e. entering a niche area after exposure to it in an American or European jurisdiction] is similar to many other legal scholars in other countries around the world. We were not exposed to the field of ‘elder law’ as such in our home countries when we studied law because the field did not exist then. However, because I entered the field as a legal scholar and an academic, and not as a practitioner, I tried to develop a more scientific approach. From a scientific approach, the perspective is not [necessarily] a practical one but also theoretical, conceptual, scientific, and methodological. Hence, my approach was to provide for a more scientific foundation to the field. So, ‘law and ageing’ is, in a sense of a legal discipline, very similar to ‘law and society’, or ‘law and economics’, or ‘law and psychology’, or ‘disability and law’, or ‘disability studies’. So, the idea was that it was not only a practice-oriented field, but rather also a scientific, methodological approach similar to other well-known scientific fields in the [broader] field of law and society. That was one idea.

The second idea was to connect the philosophy of law, meaning jurisprudence, to the field of gerontology, which is the science of ageing. Hence, ‘jurisprudential gerontology’ is the idea that we are connecting the philosophy of law, which is the science/concepts/models/theories of law, to the science/psychology/sociology of ageing. So, this shift in terminology was to provide a more scientific foundation to a legal field which is historically founded on a much more practice-oriented foundation. The importance of these concepts is to emphasise the scientific and methodological aspects of the field of ‘law and ageing’ and try to promote, advance, and move it forward beyond the very professional-like practice approach to the field.

### AG:

*I think that this answer is also very helpful to my own work, because although I have practised for a bit, most of my practice was not related to the work I do in academia, and my academic career has turned out very differently. My supervisor asked me one question before taking me on as his student; he said, ‘What is it that you want to do? Do you want to be an activist, do you want to go to courts to challenge what is happening, or do you want a theoretical framework around it?’, and I said, ‘Well, there are already challenges before courts or petitions that are being drafted but there is a very limited theoretical understanding of what the police should be doing differently.’* 27 *In that sense, I’m learning a lot from your answer even as I proceed with my own academic career.*

### IG:

Yes, and again, we need not forget that these things are interconnected. There is a limit to what you can do in practice when you do not have a theoretical foundation, but when you have that [theoretical understanding] and empirical data analysis and you have what is called evidence-based practice,^28^ the practical aspects of that field could significantly improve, be elevated, and promoted; they [theory and practice] go together hand in hand. But at least historically, and when I entered the field, while the practice was quite advanced, the theoretical foundation was non-existent, so to speak. That is why the first book I edited was called *Theories of Law and Ageing*^29^ exactly to provide, or attempt to provide for the first time, some theoretical frameworks to a field that didn’t have any prior to that.

### AG:

*This leads me to my next question. What exactly, when you were a law student, was your motivation to focus on this very niche area? Why this particular area? Was it related to something that you saw happening in Israel, or did this motivation come entirely from what you observed happening within the US?*

### IG:

There’s a famous article called ‘The Accidental Elder Law Professor’^30^ which might be relevant here. Many times, in life, you enter into fields or adopt some kind of career path purely by accident. In many ways, my decision to pick up the field of ‘law and ageing’ was by accident.

Israel, similar to India, is a country that was colonised by the British Empire,^31^ and hence we both have the common law system. When I studied law, I basically studied common law, the British common law. But this was with a twist of the human rights revolution that occurred in Israel during the late 1980s and early 1990s.^32^ It came with a belief in the idea that law can serve as an instrument for social change in general, and more specifically, that law is an instrument to promote the human rights of disadvantaged groups. In Israel, in those times, we were talking about women’s rights, rights of non-Jewish groups (for example, Muslims or Arabs) within civil society; we were talking about religious rights in the context of a country where Jews are the majority. But there was no reference to older persons, no knowledge or any awareness of older persons as a significant group within society which would need any specific attention. Broadly speaking, similar to India or Eastern traditions,^33^ there is a general notion in Judaism that there is of a lot of respect for older people, and as a very family-oriented society, the understanding of family members was that ‘we will take care of our elder members’. So, it was thought that that there is no need or interest for a specific field of ‘law and ageing’ as such.

But when I did my master’s in the US in Washington, DC in the early 1990s, I went to do an internship at an organisation called the AARP, which stands for American Association of Retired Persons,^34^ which is a lobby group that represents older persons in the US. That was the first time I was exposed to this field of ‘law and ageing’, and exposed to the sociological aspects of the demographic revolutions which I wasn’t aware of or had spent a lot of time studying about. (Through the term ‘demographic revolution’, I refer to the combination of the significant rise in life expectancy along with the decline in fertility rate, which brings—in most countries around the world—a fast and dramatic increase in both numbers and relative rate of growth of the population of older persons in the society.)

More importantly, I realised the extent of the fascinating novel developments within law which specifically address the interests of older persons. So, by pure accident and by the mere fact that while staying in the US, doing my master’s thesis, and accidentally being sent to an organisation which represents older persons, I was exposed to a whole new field that I had no awareness of prior to this. I decided that this was a whole new world and there’s always a fascination when you find a new landscape that you weren’t aware of, and then you think that that you can do a lot of new things that no one did before. Because all of that, I fell in love with this field of law and ageing and since then have attempted to flourish my scientific world within it. Personally, I always wanted to use law as an instrument for social change, and the fact that I eventually ended up doing that within the context of law and ageing was a pure accident. I can only be very thankful for that accident to have happened.

### AG:

*I will absolutely read this article you suggested because I am an accidental lawyer myself. I was initially trained as a political scientist and law happened to me much later in life, and technology law (one of my current specialisms) happened completely by accident as well, because of one of my professors at the LSE.*

*Picking up from something you said earlier, about the human rights revolution in Israel in the 1980s, I want to know about the possibility of a movement for older adults. You have written, ‘Unlike women or children, to whom the international community has given international bills of rights, there is as yet no comprehensive international convention devoted to the rights of the elderly.’* 35 *Do you feel that though there have been efforts by scholars and practitioners like yourself, there is a general lack of a broad-based movement? If yes, why do you think older adults have not been able to have a movement which is similar to feminist movements or the anti-racism movements or movements by other similarly disadvantaged groups?*

### IG:

I think there are a lot of similarities, from a historical development perspective, between the feminist movements or other civil rights movements for specific groups (like LGBTQ + persons and persons with disabilities) and the movement for the human rights of older adults. I believe these developments take time and involve a social process which touches upon the politics of identities. There is a need for older adults to develop a political sense of group identity. It took time for women to develop that identity, for persons with disabilities, for the LGBTQ + communities/movements.

The movement for the human rights of older persons, which is the most recent, is still in its early stages. Many older persons still do not have a sense of self-identity as part of the older adult groups, so to speak. On the contrary, many times, older people say that they are not old; they deny their identity as older persons. However, as we know, this does not help as it is not up to them to decide, it is what the society decides for them, and after a certain point, society treats them as older persons regardless of how they view themselves. So, yes, I totally agree that one of the reasons that unlike women and children, and persons with disabilities, older persons do not yet have an international human rights convention solely dedicated to their rights is definitely because there is a lack of a significant and powerful civil rights movement that self-identifies as an older persons’ human rights movement.

But this is a process, a social process, which I think has been pushed forward in the last two years of the COVID-19 crisis. Suddenly, all over the world, many people who denied or were blind to the fact that society viewed them as older persons realised that they were viewed or recognised or discriminated against and stigmatised as social problems solely because they were seen as old, regardless of who they are as individuals and how subjectively they view themselves. This sudden realisation of being different because you are socially constructed as old significantly contributed to the advancement of this movement, this social movement of older people realising that they need to embrace their political identity as older persons to fight against ageism. One of the ways to combat ageism is to have a unique and specific international human rights instrument that will allow them to make this social change through legal instruments. In that sense, it is similar to other kinds of civil rights movements, except it’s simply happening 30–40 years down the line. It will take time. Historically, these social changes, these movements by other social groups, took sometimes hundreds of years to make these advancements. In the context of the demographic shift,^36^ the growing numbers of older adults and, unfortunately, the growing instances of ageism they experience, and the unwillingness to accept ageist social norms will, I think, eventually bring this social movement up to the same scale as the movements for persons with disabilities or for women and children have already reached.

### AG:

*I agree with you, especially in the context of disability rights movements and how they picked up pace only recently, especially in India.* 37

*Going back to you saying that this sub-discipline of law emerged in the US, do you think that academia within the rest of the world has now caught up? There are several universities in the US which have set up entire departments or sub-departments that focus on ‘law and gerontology’.* 38 *In Germany, I could find only a few, such as the University of Heidelberg* 39 *or the University of Vechta,* 40 *that tangentially mention law with gerontology In India, only a handful of scholars here and there study ‘law and gerontology’ and there are no clinics specifically dealing with ‘law and gerontology’. Do you think that the progress in the US is being replicated in academia in other parts of the world?*

### IG:

This is a good question. I would take the optimistic side, and I will take Europe as an example. Fifteen years ago in Europe, it was almost impossible to find legal scholars who would identify themselves as experts in the field of ‘law and ageing’, and dedicated their scholarly work to this field. But in the last ten years, we have seen a significant change, and we were even able to establish a group called the ELAN (European Law and Ageing Network)^41^ where we have representatives from Sweden, Spain, France, Netherlands, and many other European countries. There is a growing number of legal scholars finding interest in this field, who are choosing to focus their scientific work on this topic. Relatively speaking, this is a new trend, so we are talking about a period spanning the last five–ten years, max.

But I can only testify for myself; it only takes one scholar who chooses to pick this topic up as a law professor and [then] you have master’s and PhD students, and then you develop a new generation of early-stage scholars who pick up this topic and then it simply grows by itself. All we need to find are leaders, and we might only need one or two in each country, so for example, in India, even if there is no leader today, as long as there is one scholar who decides that this is their field of expertise, and will then have master’s students and PhD students who will then decide to pick up this topic, in another 10, 20, 30 years, we will see a significant growth all over the world. I have another example from Australia as well, where 10–15 years ago, it was hard to find someone whose field of interest or focus was law and ageing. Today, many law schools in Australia—and I personally know various legal scholars in the country who define themselves and have chosen to focus on this field, and develop, study, and teach ‘law and ageing’ in different states in Australia. Again, this is a historical process and I am optimistic in the sense that looking forward, we will find more and more examples of courses and scholars and programmes in other countries around the world that would focus on this field.

### AG:

*Yes, fingers crossed, certainly hoping so. In India, we have at least a few books that are dedicated to gerontology (but not necessarily ‘law and gerontology’) and have been published in the last 5 years.* 42

*Coming to the books I am talking about, and the volumes that you’ve written and edited, or the authors who you may have collaborated with, have you noticed something, which has been noticed in feminist movements, and is now emerging in the disabilities rights movements as well, that there is a split between scholars who talk about ‘intersectionalities’*^43^*and those who advocate for ‘strategic essentialism’.*^44^*But because we try to find unity when there isn’t any, we also forget that certain groups might face what is called ‘double jeopardy’*,^45^*like black women who face misogyny as well as racism. Do you also think that this is or might become a problem in the scholarship on ‘law and gerontology’, or ‘jurisprudential gerontology’, where differences in gender, race, and economic status, or the Global North–Global South divide, are ignored? Do you think there is a risk that scholarship might be forgetting ‘multiple marginalisations’?*^46^

### IG:

I would like to divide my answer into two parts. Part one is that there is no doubt that today there is an understanding and awareness of the context of intersectionality, and it is very important. In gerontology, for example, for many years, the research was mostly about white older men or white older women, and the experience of ageing was portrayed as universal. But in the last five–ten years, the need to investigate the unique experiences, for example, of ageing people with disabilities, or the unique experiences of ageing LGBTQ + persons, or of even smaller groups such as the experiences of ageing prisoners, the experience of ageing while living in prisons, has risen to the point where we understand that behind these very broad descriptions and analyses of ageing or being old or older persons as a group, are hidden a lot of nuanced experiences, not only culturally, but of subgroups; for instance, the experience of poor women ageing in widowhood is very different from the ageing experience of a white privileged married woman. So, there is certainly a growing awareness of the need to investigate and focus, and be more accurate and nuanced about our understandings of the very rich and diverse experiences of ageing by subgroups and minorities.

But this touches upon broader, major tensions within any civil rights movement, between conceptual and political perspectives. If we take the feminist movements, originating from what we call the liberal approach, there is this kind of a universalistic approach that says, ‘All we want is the same rights as men.’^47^ On similar lines, this universalistic approach that says, ‘Older persons don’t want mandatory retirement, we don’t want age discrimination; treat us on an individual level and ignore our age,’ also exists within groups of older persons. Historically, we can see that the field of ‘law and ageing’ has a similar trend that originates in a very universalistic approach, equality-based approach that says, ‘We are not different, so treat us on similar grounds, do not discriminate against us in employment, in healthcare systems, etc.’ But similar to the feminist movements, we see a more advanced approach, which is a culturally oriented approach which in the case of feminism says, ‘We are not equal, women are different, women have unique aspects which we need to emphasise and stress upon.’ And so, on a similar note, within the older persons’ rights movement there is a sense that, ‘We are older people, we are different, we also have unique aspects and characteristics which we need to stress upon. We have our wisdom, our experience which needs to be reflected upon, and we don’t need to fight only for equal rights, we need to fight for unique and specific rights which reflect our uniqueness and significance as a distinct social group.’ And then you have again, similar to the feminist movements, the critical approach which says, ‘Let’s forget about age and old age as a category altogether, let’s deconstruct the whole notion of *old* or being older; age is a social construct altogether.’

You see these tensions also within the struggles for a new international human rights convention saying, ‘Well if we struggle for a unique convention, in some ways we only strengthen the ageist approach that claims that older persons are weak, different, and need special treatment; we are only strengthening this ageist construct of older people being different.’

But on the other hand, that’s the only way to move forward and older persons are different, and they do need unique and specific rights, or [we] need to tailor universalistic human rights in order to address the unique aspects of ageing. So, these are internal tensions which exist in all human rights struggles, or in all the discourse of politics of identity, about distinctions between the extent to which the politics of identity actually promotes or strengthens human rights and whether it does the opposite, which is to strengthen stereotypes and stigmas about these specific groups. These are internal tensions which exist in all fields and probably there is no one correct answer and there are arguments on both sides.

Historically, we can understand that the liberal human rights movement in many cases failed to advance the rights of the especially disadvantaged groups until the rise of identity-group politics, which was eventually successful in moving forward and making a significant social change. I think one cannot ignore this historical importance and significance of the politics of identity to be successful in changing social reality and the failure of the liberal universalistic approach to human rights to really make a difference in the rights of minority groups. So, this is an internal historical process in tension that exists as well in the field of rights of older persons.

### AG:

*I want to come to two very specific law-based questions at this point. And, one of these questions is also very close to my heart because I’m away from my family and will not be seeing them often for the next four years as my research grant restricts the amount of time I can spend back home. Several immigrants to the West are in a similar position where they cannot help their older parents emigrate and can only return to their home countries for short durations to care for those they have left behind.* 48 *You have used the term ‘distant caring’ to explain this phenomenon.* 49 *How would you critique the immigration laws of the Global North in such instances where developed countries want a skilled workforce from developing countries but fail to adequately provide for the reunion of families? As you said earlier, in Israel, and even in India, there is a tradition that expects children to look after their older parents who are often dependent on them in their later years, like my parents are. How can we deal with this dissonance between reality and these harsh immigration laws?*

### ID:

This is a tough question, and I will start by answering with a general approach. Human rights of older persons are originating from the older persons themselves, so the viewpoint is not that of the children, it’s that of the older persons. That’s point number one.

Point number two is that in my theoretical model, which is called the multidimensional model of the elder law,^50^ I have a discussion on an informal caretaker and my argument is that if we care for the rights of older persons from the perspective of the older persons themselves, we need to care for the informal networks which are providing care—meaning family members or close friends or other relatives who provide the informal care^51^—because the empirical data that we have proves that most of the care needs of older persons are provided by informal caretakers, again mostly family members, mostly women (either the wife or the daughter-in-law).^52^ So, from that sense my argument is that when we shape and reflect on laws and entitlements and rights, we also need to provide a legislative framework which provides support and assistance through this informal network which surrounds older persons and provides them care, and allows them or empowers them to maintain their human rights. I give a lot of examples of legislative initiatives, say for example, family care leave laws which allow family members to take a paid leave from their employment in order to care for the ageing parents.

Now after this broad introduction, I have to add that immigration laws are not my field of expertise and they often reflect very delicate balances between nations, all kinds of complicated national interests especially, in this very dynamic globalised world, with a lot of complex issues surrounding migration, refugees, and illegal immigration. It’s a very delicate and complicated legal reality which I am not an expert on.

Then, I want to go beyond my general argument that when shaping immigration laws, one needs to also take into account the rights of older persons within the context of, for example, the ability to provide care or receive care. For example, in Israel we had a huge wave of immigration from the former Soviet Union of Jewish people.^53^ But in some cases, their parents were left behind or in some cases the parents or grandparents were not necessarily Jewish. However, we have specific provisions which, for example, in the case of an older parent or older grandparent, even if not Jewish, provide specific rights to join their children who live in Israel in order for them to provide care. Again, I cannot say something more specific, but I do agree that when tailoring legislation in rights around immigration or migration, one does need to take into account issues of care and intergenerational care in order to acknowledge the rights of older persons to receive care by their family members. How to exactly do it is a delicate and complicated field of law which needs more attention, and I haven’t paid that attention yet.

### AG:

*Your idea about paid family care leave makes a lot of sense because I don’t have that. Perhaps, if the immigration laws are too complex to be overhauled, at least regulations for the skilled workforce that emigrates to the developed world can be tailored to give more leeway for them to go back home and care for the family members they’ve left behind; maybe longer (in my case, longer than six weeks) paid family care leave can be considered as a relatively less controversial measure.*

### ID:

That’s a great idea and I haven’t thought about it a lot, but now that you are raising this idea, I totally agree, and I think that it’s a great idea to promote and advance these reforms in immigration laws to allow exactly that. Yeah, I fully agree.

### AG:

*This leads me to another very specific law-based question, and this is a developing issue in India. Ethically and morally, we have been having this discussion for generations and it is now entering the legal domain as well*—*about sanctions against the abandonment of ageing parents, or neglect/abuse of ageing parents. It is morally frowned upon, socially frowned upon, but legally there were not many penal provisions for parents, except Section 125 of the Code of Criminal Procedure 1973 that provides for parents to move courts, demanding that their children pay them a monthly maintenance. Failure to comply with the court order may entail imprisonment (albeit for a short duration of one month). But now, the Indian central government has proposed changes to the Maintenance and Welfare of Parents and Senior Citizens Act 2007 that would punish the abuse and abandonment of ‘senior citizens’.*^54^*Some other legal systems may not treat the abandonment of older adults in the same way as India (because the moral disapproval of abandonment, which is prevalent in India, may not be so prominent there) but do have penal provisions for domestic violence against older adults. The Domestic Abuse Act 2021 in the United Kingdom contains no upper age limit for a person to be classified as a ‘victim-survivor’ of domestic abuse, thereby covering older adults.*^55^*Several states in the US have also criminalised the abuse of older adults.*^56^*Professor Nina Kohn has been critical of this criminalisation, drawing from anti-carceral feminism.*^57^*Do you think that a criminal justice response to violence against older persons is useful?*

### ID:

One needs to differentiate between two totally different elements of your question. The first one, which is easier for me to address, is the phenomenon of elder abuse and neglect. Unfortunately, this is a very well-known phenomenon and includes all aspects of abuse—not just physical, mental, and financial, but also neglect (both active and passive). There is a whole field of elder abuse and neglect, and there is legal literature on how to address these issues,^58^ to what extent criminal laws are sufficient or enough, mandatory reporting, criminalisation and other legal avenues to address and combat elder abuse and neglect. So, this is one aspect to the question. Generally speaking, there is growing empirical and conceptual literature with these various approaches. I think there is a growing understanding that criminal law, and criminalisation, is only one answer but it cannot be the sole answer; only criminalisation and punishment will not solve the issue. There are many other aspects that need to be taken into account in order to try to alleviate this, similar to other complicated social issues like violence against women or abuse of children. None of these complicated social phenomena can be resolved solely through criminalisation.

The other aspect, which in my view is more complicated, is the issue of abandonment. To what extent should we and can we legally bind adult children to care for their parents and to what extent do we expect adult children to care for their parents? Now, on this topic there was a historical development, because if you look at the ways in which laws in the western world addressed poverty, the traditional Elizabethan poor laws were based on local and family responsibility laws;^59^ if there was an older person who was poor, let their family take care of them, and it was the responsibility of families to care for the older parents. The old Abrahamic religions placed this sense of moral duty upon families to provide for the physical and financial care of their older parents; it was inherent in moral duties.

But the development of the welfare state and the social rights movement was aimed at, to some extent, providing an individual social security at all stages of life, including old age, regardless of one’s family. The idea of the modern welfare state is that when you become old—in theory like any other dependency situations, for example, when you become disabled, or you become sick—the welfare state needs to ensure and provide you a safety net which makes you autonomous and not dependent on your children, which is true in all fields of life. From a perspective of social rights or welfare rights, it is the duty of the society to ensure that every older adult at any age, for example a woman at the age of 95, be able to live independently, financially speaking, as the state will provide her, for example, a sufficient old age pension which can make her live reasonably and not in poverty, without being dependent on the financial support of or to rely on the goodness of her children.

On the other hand, there is still a notion that filial piety and filial responsibility are still a valid moral rule. And there is still a sense that the children do need to provide for their parents. This tension is still evident many countries, and the question is how you legislate it or how you legally construct it. Personally, as a welfare state supporter and from the human rights perspective of the older person themselves, my approach is that it is the duty of the state to provide a system of social security and social rights which ensure a minimal safeguard for every older person to live with dignity and not in poverty, by themselves, regardless of how their children treat them. From my perspective, the informal care network and the duty of children to provide and care for their parents is a moral duty, a religious duty, a norm, but this is not something that we can build in a legal system to ensure the rights of a person, based on the duty of the children to take care of them; it is the duty of the state and society, like many other social rights like the right to health, right to transportation, right to education. They need not be dependent on their adult children; it is within and by itself a right of the older persons, and the duty of the state. However, we still need to shape and provide legislations—as I mentioned earlier—to support, to enable, to allow for those family members to encourage, and to continue to care for older persons. But the prevention of poverty as a policy in human rights need not be founded or based on the mandate that children should provide. That’s my personal approach.

### AG:

*Thank you so much for this nuanced response, especially the delineation of this distinction between abuse/neglect and abandonment.*

*My next questions relate specifically to the pandemic. You have spoken about identity creation being accelerated during the pandemic and COVID-19 acting as a catalyst. In the initial stages of the COVID-19 pandemic, several countries introduced certain protectionist measures for older adults. For instance, they were required to undergo mandatory hospitalisation if they tested positive, longer periods of social isolation even though cases were waning, temporary closure of care homes, and so on.* 60 *Do you think that such protectionism was the best way to mitigate the challenges posed by COVID-19, considering that it aggravated already elevated levels of loneliness and isolation amongst retired adults?* 61 *We have a new spectre haunting us—the WHO has declared monkeypox as the newest global health emergency. Do you think legal systems around the world are better prepared to account for the psychosocial needs of older adults going forward; have lessons been learnt?*

### ID:

First, I fully agree with you. I think, again as I mentioned earlier, that the last two years have provided really clear evidence of how strong and rooted ageism still is in society, and now under the guise of protectionism, as you defined it, we continue to discriminate against and infringe the human rights of older persons. I totally agree with you that the case of the last two years proves the need for a paradigm shift in the way we approach policies towards older persons—from a paternalistic and protectionist approach to a human rights–based approach. This relates to what I mentioned earlier, the realisation of many older adults and the rise of this activist behaviour of older adults who refuse to accept these kinds of policies and behaviours any longer.

Have we learnt the lesson? This is a more complicated question. Humankind’s memories are quite short, unfortunately, and the struggle is ongoing. In this sense, nothing can be taken for granted and the struggle to continue to advance, promote, advocate for the rights of older persons, and combat ageism is an ongoing long process. I am not sure if we have learnt the lesson. For example, we still don’t have an international convention for the rights of older persons, and at least from my sense in the recent open-ended working group meeting at the United Nations,^62^ there is still not a political breakthrough to move forward quickly on that front. So, like other social movement struggles, this is an ongoing, long, and hard process in which COVID-19 was a trigger, and at least I hope, or would like to wish, that it will make a significant change in the rhetoric, behaviour, and discourse, and in the activity and activism of older persons themselves, and their sense of political identity. But will it be successful? Will, in the next pandemic, reality be very different? I am not sure, I don’t know. It will be up to us, and it will be up to the activism that would need to continue all the time. I don’t know, it would be interesting to see, but one thing I know for sure is that we cannot take anything for granted. It’s not as if, ‘Okay, we have had COVID, we have learnt the lesson, next time things will be much better, we won’t see these policies, things will be different.’ I don’t think this will be the case; if we do not work hard and continue to work hard, things will not change.

### AG:

*We now come to our last question that I would like to connect to your point about activism. In an interview from June 2020, when perhaps we hadn’t yet seen the worst of the pandemic, you spoke about how older adults themselves were fighting battles against these discriminatory rules and mandates that governments were coming up with. Could you tell us about the work that you did then as the Chairperson of the Association of Law in the Service of the Elderly? What role did this association play in Israel’s response to COVID-19? Do you think that governments at different levels were responsive to the suggestions made by civil society actors such as yourself and the people you call have called ‘ageivists’?* 63

### ID:

Let me split my answer into two. Law in the Service of the Elderly is an association that I created 22 years ago; it’s based on a classic ‘law as an instrument for social change’ approach. The instrument we use is ‘strategic litigation’;^64^ we take cases to the Supreme Court and through the Supreme Court we presume or assume that the reality will change. Specifically in the context of COVID-19, we took a case to the Supreme Court regarding a rule that discriminated against older workers after the lockdown; younger workers were allowed to return to work but older workers were not. This was very similar to the example that you gave. So, we took it to the Supreme Court, and fortunately, the government changed the rules and there was no need for a decision by the Supreme Court. Law is an instrument for social change and strategic litigation is only one tool in a broader box of tools.

I think that what we need more is what I call ‘age-ivism’^65^ or the activism of older persons themselves; not from courts, but from traditional activism. It can be a demonstration, it can be a political lobby, it is being active on the streets, or in the media or the digital world. It includes all fronts of social activism that we historically and traditionally know, and new forms of social activism that are now available from new technology and the digital world. This needs to come from older persons themselves.

One of the most impressive elements of the disability rights movements was the slogan ‘Nothing About Us Without Us’,^66^ and I think this notion of visibility of persons with disabilities, which was very evident in their movements, needs to be duplicated and be there in the context of older persons’ movements. The visibility of leaders who are themselves older persons—and identify themselves as older person, and are proud of themselves as older persons, and are activists in these struggles and movements—is very important. It is not enough to have a Supreme Court case. It is important, but that in and by itself will not lead to a social change. That is what I mean by ‘age-ivism’; an activist movement for older persons, by older persons is very important.

I would like to conclude with thanking you for giving me this opportunity to share my views and experiences in the field of law and ageing.

## Footnotes

1 Indian Public Health Association, Indian Association of Preventive and Social Medicine, and Indian Association of Epidemiologists, ‘Full Text: Draconian Lockdown, Incoherent Strategies Led to India Paying a Heavy Price, Say Experts’ (*Scroll.in*, 31 May 2020). https://scroll.in/article/963384/full-text-draconian-lockdown-incoherent-strategies-led-to-india-paying-a-heavy-price-say-experts. Accessed 23 September 2022.

2 Smriti Mallapaty, ‘The Coronavirus Is Most Deadly If You Are Older and Male — New Data Reveals the Risks’ (*Nature*, 28 August 2020). https://www.nature.com/articles/d41586-020-02483-2. Accessed 23 September 2022.

3 I tried looking for Delhi government orders that would provide a legal basis for this mandatory hospitalisation but could find none. The only relevant order I could find from back then is dated 13 November 2020 which simply directs health teams to visit patients under home isolation. See http://health.delhigovt.nic.in/wps/wcm/connect/48e8d38040815b59ac1dbca50c073453/ord16nov.pdf?MOD=AJPERES&lmod=2047575562&CACHEID=48e8d38040815b59ac1dbca50c073453. Accessed 23 September 2022.

4 Veronika Bilkova, ‘COVID-19 and Older Persons—In Need of a Comprehensive Human Rights Approach’ (2022) 14(1) *Journal of Human Rights Practice* 1.

5 Sarah Docherty et al., ‘The Effects of COVID-19 Lockdown on Health and Psychosocial Functioning in Older Adults Aged 70 and Over’ (2021) 7 *Gerontology & Geriatric Medicine* 1; Sarah De Pue et al., ‘The Impact of the COVID-19 Pandemic on Wellbeing and Cognitive Functioning of Older Adults’ (2021) 11 *Scientific Reports* 4636.

6 Srinivas Goli, et al., ‘Economic Independence and Social Security among India’s Elderly’ (2019) 54(39) *Economic and Political Weekly* 32.

7 The said article reads as follows: ‘41. *Right to work, to education and to public assistance in certain cases.*—The State shall, within the limits of its economic capacity and development, make effective provision for securing the right to work, to education and to public assistance in cases of unemployment, old age, sickness and disablement, and in other cases of undeserved want.’

8 Martha Albertson Fineman, ‘“Elderly” as Vulnerable: Rethinking the Nature of Individual and Societal Responsibility’ (2012) 20(1) *Elder Law Journal* 71.

9 Ibid.

10 Ibid. 84.

11 Ibid. 85.

12 Israel Doron, ‘From National to International Elder Law’ (2005) 1 *Journal of International Aging, Law & Policy* 43, 50.

13 https://www.facebook.com/groups/IndianinBerlin/. Accessed 27 November 2022.

14 Sections 29, 36, and 36a of the German Residence Act in the version promulgated on 25 February 2008 (*Federal Law Gazette* I, p. 162), most recently amended by Article 4b of the Act of 17 February 2020 (*Federal Law Gazette* I, p. 166), only facilitate the emigration of the spouse of the foreigner, children of the foreigner aged under 18, and parents of those with German residence permits aged under 18. Only family members can be granted a temporary residence permit in cases of exceptional hardship.

15 Section 51 subsection (1) No. 7 of the Residence Act mentioned in the previous footnote.

16 TK Rajalakshmi, ‘Omicron Cases Fuelling the Surge in India’s Third COVID-19 Wave’ (*Frontline*, 28 January 2022). https://frontline.thehindu.com/covid-19/omicron-cases-fuelling-the-surge-in-india-third-covid19-wave/article38189259.ece. Accessed 24 September 2022.

17 Part 1.5.4 in DAAD, *Your DAAD Scholarship: Guidelines and Information* (German Academic Exchange Service 2022) 20.

18 Michelle Putnam, ‘Replacing *the Elderly* with *Older Adults* in JGSW Publications’ (2015) 58(3) *Journal of Gerontological Social Work* 229; Dale Avers et al., ‘Editor’s Message: Use of the Term “Elderly”’ (2011) 34(4) *Journal of Geriatric Physical Therapy* 153.

19 Jere Longman, ‘85-Year-Old Marathon Runner is So Fast that Even Scientists Marvel’ (*New York Times*, 28 December 2016). https://www.nytimes.com/2016/12/28/sports/ed-whitlock-marathon-running.html#:~:text=It%20is%20not%20his%20arm,miles%20in%20under%20four%20hours. Accessed 24 September 2022.

20 See for instance, Gayle Kimball, *Ageism in Youth Studies: Generation Maligned* (Cambridge Scholars Publishing 2017).

21 For example, the Hebrew word *kashish*, which is commonly used to describe older persons, implies that the person is weak, frail, and vulnerable.

22 Israel Doron and Helen Meenan, ‘Time for Geriatric Jurisprudence’ (2012) 58 *Gerontology* 193.

23 Israel Doron, ‘Law, Gerontology, and Human Rights: Can We Connect Them All?’ (*OUPBlog*, 20 May 2013). https://blog.oup.com/2013/05/law-gerontology-human-rights/. Accessed 24 September 2022.

24 I am calling these terms ‘commonly used’ on the basis of a cursory search within the library catalogue of my alma mater, the London School of Economics and Political Science; the library shows 207,601 articles on elder law and 965 books, and only 15 articles and no books containing the phrase ‘geriatric jurisprudence’.

25 Deb Hipp, ‘Hiring an Elder Law Attorney’ (*Forbes Health*, 23 June 2022). https://www.forbes.com/health/healthy-aging/elder-law-attorney/. Accessed 24 September 2022.

26 Ibid.

27 As a PhD scholar, my research broadly centres around the role of policing in a data-driven world.

28 See Liz Trinder and Shirley Reynolds (eds), *Evidence-Based Practice: A Critical Appraisal* (Blackwell Science 2000) for a more detailed definition.

29 Israel Doron (ed), *Theories on Law and Ageing: The Jurisprudence of Elder Law* (Springer-Verlag 2009).

30 A Kimberley Dayton, ‘The Accidental Elder Law Professor’ (2010) 40 *Stetson Law Review* 97.

31 Charles Glass, ‘The Mandate Years: Colonialism and the Creation of Israel’ (*Guardian*, 31 May 2001). https://www.theguardian.com/books/2001/may/31/londonreviewofbooks. Accessed 24 September 2022.

32 See Aharon Barak, ‘Human Rights in Israel’ (2006) 39(2) *Israel Law Review* 12.

33 Kyu-taik Sung, ‘Elder Respect: Exploration of Ideals and Forms in East Asia’ (2001) 15(1) *Journal of Ageing Studies* 13.

34 https://www.aarp.org/ Accessed 04 November 2022.

35 Doron, ‘From National to International Elder Law’ (n 12) 58.

36 Department of Economic and Social Affairs, Population Division, *World Population Ageing 2019* (United Nations 2019) 1.

37 Ankita Gandhi and Surabhi Singh, ‘Disability Studies and the Law: In Conversation with Professor (Dr) Nilika Mehrotra’ (2021) 12(2) *Jindal Global Law Review* 433.

38 See, for example, Stetson University (https://lawblog.law.stetson.edu/mj/what-exactly-is-an-aging-law-policy-degree. Accessed 04 November 2022), Nebraska College of Law (https://law.unl.edu/joint-degree-gerontology/. Accessed 04 November 2022), Syracuse University (https://asi.syr.edu/. Accessed 04 November 2022).

39 https://www.uni-heidelberg.de/en/study/all-subjects/gerontology-health-and-care. Accessed 04 November 2022.

40 https://www.uni-vechta.de/en/gerontology. Accessed 04 November 2022.

41 https://law.lu.se/webukv2.nsf/menuItem.xsp?SessionID=F25FB2BE63C84B839AFA7CBC975CAB9263E4DC94#!elderlaw-elan. Accessed 04 November 2022.

42 See for instance Mala Kapur Shankardass (ed), *Ageing Issues in India: Practices, Perspectives and Policies* (Springer 2021); S Irudaya Rajan and Gayathri Balagopal (eds), *Elderly Care in India* (Springer 2017).

43 To read more about the meaning of ‘intersectionality, see Jane Coaston, ‘The Intersectionality Wars’ (*Vox*, 28 May 2019). https://www.vox.com/the-highlight/2019/5/20/18542843/intersectionality-conservatism-law-race-gender-discrimination. Accessed 24 September 2022.

44 ‘Strategic essentialism’ was a term introduced (and later partially disavowed) by postcolonial scholar Gayatri Chakravorty Spivak. See Kristina Wolff, ‘Strategic Essentialism’ in George Ritzer (ed), *The Blackwell Encyclopedia of Sociology* (Blackwell Publishing 2007).

45 Frances M Beal, ‘Double Jeopardy: To Be Black and Female’ in Robin Morgan (ed), *Sisterhood Is Powerful: An Anthology of Writings from the Women’s Liberation Movement* (Vintage Books 1970).

46 Clary Krekula, Pirjo Nikander, and Monika Wilińska, ‘Multiple Marginalizations Based on Age: Gendered Ageism and Beyond’ in Liat Ayalon and Clemens-Tesch-Römer (eds), *Contemporary Perspectives on Ageism* (SpringerOpen 2018); Colin Duncan and Wendy Loretto, ‘Never the Right Age? Gender and Age-Based Discrimination in Employment’ (2004) 11(1) *Gender, Work & Organization* 95.

47 AR Baehr, ‘Liberal Feminism’ (*Stanford Encyclopedia of Philosophy*, 18 October 2007). https://plato.stanford.edu/entries/feminism-liberal/. Accessed 24 September 2022.

48 For the UK, see https://www.gov.uk/uk-family-visa/adult-dependent-relative. Accessed 04 November 2022. Emigrating parents need to show that the long-term care to do everyday tasks because of disability or age, care they need, is not available or affordable in their own country, and their children can support them without claiming public funds in the UK.

49 Doron, ‘From National to International Elder Law’ (n 12) 50.

50 Israel Doron, ‘A Multi-dimensional Model of Elder Law: An Israeli Example’ (2003) 28(3) *Ageing International* 242.

51 For a definition of ‘informal care’, see Jia Li and Yajun Song, ‘Formal and Informal Care’ in Danan Gu and Matthew E Dupre (eds), *Encyclopedia of Gerontology and Population Aging* (Springer Cham 2020).

52 Athina Vlachatoni and Maja Fuglsang Palmer, ‘Gender and Caring in Later Life’ in Danan Gu and Matthew E Dupre (eds), *Encyclopedia of Gerontology and Population Aging* (Springer Cham 2020).

53 Elazar Leshem and Moshe Sicron, ‘The Absorption of Soviet Immigrants in Israel’ (1999) 99 *American Jewish Yearbook* 484.

54 Section 2(h) of the Act defines a ‘senior citizen’ as one who has attained the age of 60 years.

55 Louise Shepperd, ‘Domestic Abuse of Older People’ (*UK Parliament: House of Lords Library*, 09 July 2021). https://lordslibrary.parliament.uk/domestic-abuse-of-older-people/. Accessed 24 September 2022.

56 Chapter 13 of Title 9 (Section 368) of the California Penal Code. Nevada, Idaho, and Oklahoma also have similar laws.

57 Nina A Kohn, ‘Elder (In)Justice: A Critique of the Criminalization of Elder Abuse’ (2012) 49(1) *American Criminal Law Review* 1. Kohn argues that criminalisation of elder abuse, like the criminalisation of domestic violence, often wrests control away from the survivor and places it in the hands of the state. To explain this point, she cites examples of laws for the arrest and prosecution of alleged abusers even though the survivor may not be in favour of such forms of redressal and may not see them as ‘remedies’. This gives more power to an already pervasively dominant state at the expense of the survivor, replacing one system of social domination by another.

58 See A Lowenstein and I Doron, ‘Times of Transition: Elder Abuse and Neglect in Israel’ (2008) 20(2) *Journal of Elder Abuse and Neglect* 181.

59 Seymour Moskowitz, ‘Filial Responsibility Statutes: Legal and Policy Considerations’ (2001) 9(3) *Journal of Law and Policy* 709.

60 Bilkova, ‘COVID-19 and Older Persons’ (n 4); Agence France-Presse, ‘EU Chief: Social Isolation for Elderly May Last until Year’s End’ (*VOA*, 12 April 2020). https://www.voanews.com/a/science-health_coronavirus-outbreak_eu-chief-social-isolation-elderly-may-last-until-years-end/6187383.html. Accessed 24 September 2022.

61 Docherty et al., ‘The Effects of COVID-19 Lockdown on Health and Psychosocial Functioning in Older Adults’ (n 5); De Pue et al., ‘The Impact of the COVID-19 Pandemic on Wellbeing and Cognitive Functioning of Older Adults’ (n 5).

62 12th Open-Ended Working Group on Ageing for the Purpose of Strengthening the Protection of the Human Rights of Older Persons (11 to 14 April 2022). https://social.un.org/ageing-working-group/twelfthsession.shtml. Accessed 24 September 2022.

63 Ellie Parravani, ‘How a Coalition of Organisations Challenged Ageism in Israel’s Response to COVID-19’ (HelpAge International, 12 June 2020). https://www.helpage.org/newsroom/latest-news/how-a-coalition-of-organisations-challenged-ageism-in-israels-response-to-covid19/. Accessed 24 September 2022.

64 According to the European Center for Constitutional and Human Rights, ‘strategic litigation’ means the use of law and courts to effect change that has not been addressed by politics and policy. https://www.ecchr.eu/en/glossary/strategic-litigation/. Accessed 24 September 2022.

65 Israel Doron, ‘Re-thinking Old Age: Time for Ageivism’ (2018) 27(1) *Human Rights Defender* 33–35.

66 As explained by Gandhi and Singh, ‘In the context of the disability rights movement, author and scholar James Charlton attributes the origin of the slogan “Nothing About Us Without Us” to South African disability rights activists Michael Masutha and William Rowland, who had in turn heard it being used by someone at an international disability rights conference. The slogan is a demand for control, which as per Charlton appeals to the disability rights movement “because the needs of people with disabilities and the potential for meeting these needs are everywhere conditioned by a dependency born of powerlessness, poverty, degradation, and institutionalisation.”’ James I Charlton, *Nothing About Us Without Us: Disability Oppression and Empowerment* (University of California Press 2000) 3, cited by Gandhi and Singh, ‘Disability Studies and the Law’ (n 37) 445.

